# Peter Gouras, MD 1930–2021

**DOI:** 10.1007/s00417-021-05303-z

**Published:** 2021-07-30

**Authors:** Daniel J. Salchow

**Affiliations:** Charité - University Medicine Berlin, Berlin, Germany

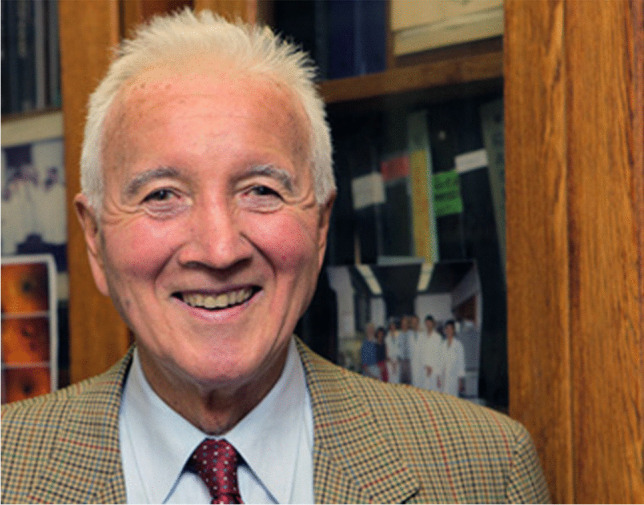



“I can put you up at our house until we find a local student room.” It was Peter Gouras, MD, who offered me a roof over my head and seat at his table, when I first came to New York. I had been invited to do research at his lab in 1997. As a young medical student from Germany, I was somewhat nervous about the Bronx address (I did not know that the house was at the most beautiful part of the Bronx, Riverdale, overlooking the Hudson River). Arriving by train at the local station in the evening, I walked up to a stately old mansion and, glancing through one of the high windows, I saw a man sitting at the dinner table. He answered the door and invited me to share the meal he had prepared. This is how I met Peter in 1997, and my first impression was that of a generous, humorous and deeply cultured man, blessed with one of the sharpest minds I have ever met. Peter Gouras died on January 8, 2021, age 90.

What could a medical student learn from this professor of ophthalmology and genuine New Yorker gentleman? Just about everything there was to learn. Being married to a German, Peter was able to read German newspapers and spoke our complex language very well. “Deutsch ist nicht meine Muttersprache, aber meine Schwiegermuttersprache.” was how he described it (“German is not my mother tongue, but my mother-in-law’s tongue.”) He and his family welcomed me warmly as they had done many times before and did many times after, making it easier for young researchers to enter the scientific world without feeling lost on a personal level.

Peter was born in Brooklyn, NY, in 1930. From his background, it was all but likely he would end up as a world-renowned vision researcher. His parents, Greek and Irish as far as I know, did not come from wealth and did not have an academic background. Nevertheless, endowed with exceptional curiosity and intelligence, Peter attended some of the most respected institutions in the USA and received his MD from Johns Hopkins, Baltimore.

I have rarely seen a scientist as enthusiastic and energetic about his work as Peter. When he identified a problem, he attacked it with all the instruments at his disposal. Even a Thanksgiving dinner could then be considered “a waste of time”, because it could be spent in the lab, making new discoveries. After having worked at the Ophthalmology Section of the National Institute of Health (NIH) in Bethesda from 1970 to 1978, he had moved back to New York when this city was considered quite dangerous. In fact, he had some frightening stories to tell from these rough years in the “Big Apple”. During one of my research periods at the Gouras lab on the 4^th^ floor of the Harkness Eye Institute, College of Physicians & Surgeons, Columbia University, New York, I remember reading a line of an interview Peter had given about how science should be conducted: “Ask the biggest possible question with the techniques you have mastered.” And he had mastered many techniques and asked major questions in vision research. In some areas, he had become a pioneer. For Peter, science was fun and “It is like talking to god,” was his perspective. The narrative of his discussion with the creator left a remarkably productive paper trail. PubMed lists close to two hundred scientific papers published by Peter Gouras, in addition to the uncounted lectures and presentations he gave at meetings and while visiting colleagues around the world. He also served as a reviewer and was active on several scientific boards.

At his lab, I encountered an inspiring group of people from all over the world and of just about all ages. The discrepancy between the outdated equipment on the one hand and the bustling scientific activity led by this genius on the other was striking. Being accustomed to well-insulated windows and functioning light switches, I learned that you do not need either to conduct world-class research. Peter investigated whatever the eye, particularly the retina, had to offer to the astute scholar. He measured electrical activity, transplanted retinal cells, worked on vitamin A metabolism and thus made numerous original discoveries and contributions to visual science. What I found most impressive was how he was able to apply basic concepts to the concrete problem at hand.

Peter was noticeably askance, when he had identified a flaw in a research hypothesis. He did not mean this in a personal way, although his frankness was occasionally perceived as harshness. He was exquisitely fair and accepted convincing arguments from anyone, including students and novices in the scientific arena. Statistics were met with profound skepticism. For Peter, a finding had to be obvious and striking to be significant, because as he told me “statistics can prove just about anything you want.”

Apart from his work, Peter had a deep knowledge of history, biology, politics and many other areas of life. He enjoyed music, good food and drink just as much as he did social events (the St. Patrick’s Day Parties at his lab were legendary).

It is with deep gratitude that I remember this unique gentleman and researcher. His gift of enthusiasm for his work and scientific vigor inspired several generations of young doctors, some of whom became leaders in ophthalmology themselves. His generosity extended beyond his work and family to reach many. Peter Gouras died age 90 in Germany, the country he had visited many times. He will be remembered by those of us who had the good fortune to meet him.

